# Genome-Wide Identification and Expression Analysis of the Sweet Cherry Whirly Gene Family

**DOI:** 10.3390/cimb46080474

**Published:** 2024-07-26

**Authors:** Lili Wang, Qiandong Hou, Guang Qiao

**Affiliations:** 1 Guizhou Academy of Agricultural Sciences, Guiyang 550025, China; smartkkk2002@163.com; 2 Key Laboratory of Plant Resource Conservation and Germplasm Innovation in Mountainous Region (Ministry of Education), College of Life Sciences/Institute of Agro-Bioengineering, Guizhou University, Guiyang 550025, China; qiandhou@163.com

**Keywords:** Whirly gene family, sweet cherry, abiotic stress, expression, promoter analysis

## Abstract

Sweet cherry (*Prunus avium*) is one of the economically valuable horticultural fruit trees and it is widely cultivated throughout the world. Whirly (WHY) genes are a unique gene family with few members and have important biological functions in plant growth, development, and response to abiotic stress. This study utilized whole-genome identification to conduct a comprehensive analysis of the WHY genes in sweet cherry and examined their transcription levels in different tissues and under abiotic stress to explore their functions. Two WHY genes were identified in the sweet cherry genome and named *PavWHY1* and *PavWHY2*, respectively, based on their homology with those in *Arabidopsis thaliana*. Both genes have theoretical isoelectric points greater than seven and are hydrophilic proteins, suggesting that they may be localized in plastids. The two genes are evolutionarily classified into two categories, with large differences in gene structure, and highly similar protein tertiary structures, and both have conserved domains of WHY. *PavWHY1* and *PavWHY2* are collinear with *AtWHY1* and *AtWHY2*, respectively. The promoter sequence contains *cis*-acting elements related to hormones and abiotic stress, which are differentially expressed during flower bud differentiation, fruit development, and cold accumulation. qRT–PCR showed that *PavWHY1* and *PavWHY2* were differentially expressed in flower and fruit development and responded to low temperature and exogenous ABA treatment. The recombinant plasmid pGreenII-0800-Luc with the promoters of these two genes can activate luciferase expression in tobacco. Protein interaction predictions indicate that these gene products may interact with other proteins. This study reveals the molecular features, evolutionary relationships, and expression patterns of sweet cherry WHY genes, and investigates the activities of their promoters, which lays the foundation for further exploration of their biological functions and provides new insights into the WHY gene family in Rosaceae.

## 1. Introduction

Sweet cherry (*Prunus avium* L.) is a horticultural fruit tree with very high economic value that is widely grown all over the world. The global annual production of sweet cherry is around 2.2 million tons, and the total planted area in China reaches 233,300 ha, with an output of more than 1.2 million tons [1]. However, the traditional breeding cycle of sweet cherry varieties is long, so gene selection is one of the current strategies to speed up the breeding cycle [1]. However, sweet cherry is greatly affected by temperature during growth, especially spring frosts and variable temperatures during spring flowering [2]. Appropriate temperatures are essential for proper plant growth and development and are indispensable in the agricultural production process, being one of the prerequisites for higher yields. Extreme temperature as a kind of abiotic stress is studied in many plants, including in terms of gene expression, molecular regulation, and changes in physiology and biochemistry [3,4]. Many genes are involved in plant growth in response to changes in the external environment, such as WRKY [5], ARF [6], DOF [7], and Whirly [8].

The Whirly (WHY) gene family consists of a relatively small number of transcription factors unique to plants, playing essential roles in DNA recognition, replication, and telomere maintenance, as well as in the maintenance of nuclear and organelle genomes [9]. This family member has a conserved whirligig secondary structure and contains a typical KGKAAL motif in the DNA-binding structural domain [10]. WHYs are highly conserved in angiosperms, and some similar proteins may exist in algae [10,11]. The first WHY protein identified was *PBF-2* from potato (*Solanum tuberosum*), which preferentially recognizes and binds to the single-stranded form of the elicitor response element (ERE) and was named *StWhy1* in later studies [12,13]. The protein tertiary structure indicates that the four *StWhy1* form a rotationally symmetric structure that is also present in other plants [12]. The β-fold forms blade-like extensions that protrude from the α-helix core, producing a swirly aspect. The tetramer is stabilized by a C-terminal helix–loop–helix motif, with numerous residues forming multiple contacts between individual proteins [12].

The Whirly gene family has few members, with only 3 members present in *Arabidopsis thaliana* [14], although it may have more members in some polyploid plant species, such as 10 genes in alfalfa (*Medicago sativa* L.) [15]. Moreover, more and more members of the Whirly gene have been identified in plants using genome-wide identification. In most plants, there are only two members of Whirly. Two of the three members (AtWHY1, AtWHY2, and AtWHY3) in *Arabidopsis thaliana* are localized to chloroplasts and one is localized to the mitochondria [16]. It has also been shown that AtWHY1 is present in both the nucleus and plastids and may carry out transmembrane signaling [14]. *AtWHY2* was found to be associated with mitochondrial DNA, and aberrant expression of this gene impedes mitochondrial formation and development [17]. Overexpression disrupts mitochondrial function by causing a decrease in transcript levels and mtDNA content, with effects on the presence of complexes on the respiratory chain thereby reducing activity [17]. The *AtWHY3* and *AtWHY1/3* double mutants showed a significant delay in flowering, with more siliques per plant but fewer seeds per silique compared with the WT [18]. It was found that WHY genes have a key role in defense response, and *StWHY1* in potato is a direct homologue of *AtWHY1* in *Arabidopsis thaliana*, whose binding activity to DNA is directly induced by salicylic acid, which exerts a biological function in antiviral processes [12,19].

Genome-wide analysis of a gene family is a method to explore the function of the gene, and progress has been made in many gene families and different plant species. The external environmental influences on sweet cherry during growth and development have always been a scientific problem worth exploring, especially in gene regulation. The analysis and functional characterization of some gene families of sweet cherry are available, but the biological functions of sweet cherry WHYs have not been explored. Because of the important function of WHYs genes in plants, it is hypothesized that WHY genes also have similar functions in sweet cherry, so the identification and analysis of the sweet cherry WHY gene family is necessary. In addition, since sweet cherries are mostly grown in facilities, such as rain shelters, which may affect photosynthesis or respiratory functions, and WHYs genes have biological functions in plastids or mitochondria, an in-depth study of the WHY gene family in sweet cherries could contribute to the understanding of these processes. Based on the functions of WHY genes, the present study intends to perform a genome-wide characterization of WHY gene members and a systematic analysis of the molecular features, gene structures, evolutionary relationships and expression patterns of these members. Furthermore, the promoter activity of sweet cherry WHY is also explored to analyze its potential biological functions. This study can provide a reference for the breeding of sweet cherry excellence and lay a foundation in screening potentially valuable molecular regulatory signaling studies.

## 2. Materials and Methods

### 2.1. Genome-Wide Whirly Gene Members Identification

Genome-wide identification and analysis of gene family members was performed with reference to previous studies [20]. For details, the sweet cherry “Tieton” V2.0 genome was downloaded from the GDR database (https://www.rosaceae.org/ (accessed on 16 July 2024 Beijing time)), and the WHY members in *Arabidopsis thaliana* and rice were obtained from PlantTFDB (https://planttfdb.gao-lab.org/ (accessed on 12 July 2024 Beijing time)). The protein sequences in the sweet cherry genome were blasted using all WHY members in rice and *Arabidopsis thaliana*; after obtaining the members (e < 10^−20^), the conserved structural domains were identified in NCBI (https://www.ncbi.nlm.nih.gov/cdd/ (accessed on 12 July 2024 Beijing time)). After obtaining the final gene members, homologous genes were named by alignment with WHY in *Arabidopsis thaliana*. Molecular weight (MW), theoretical isoelectric point (pI) and grand average of hydropathicity (GAH) of these members were calculated in TBtools v2.096 [21]. The subcellular localization of the proteins was predicted in DeepLoc-2.0 (https://services.healthtech.dtu.dk/services/DeepLoc-2.0/ (accessed on 9 July 2024 Beijing time)), and the two highest scores were obtained for collation.

### 2.2. Phylogenetic Tree, Gene Structure and Conserved Motifs

Members of WHYs from *Arabidopsis thaliana*, rice (*Oryza sativa*), tomato (*Solanum lycopersicum*), apple (*Malus × domestica*), peach (*Prunus persica*), wheat (*Triticum aestivum*), thistles alfalfa (*Medicago sativa*), plum (*Prunus salicina*), and maize (*Zea mays*) were obtained in PlantTFDB, sequence-aligned using CLUSTALW online software (https://www.genome.jp/tools-bin/clustalw (accessed on 11 July 2024 Beijing time)), and visualized in ESPript (https://espript.ibcp.fr/ESPript/cgi-bin/ESPript.cgi (accessed on 11 July 2024 Beijing time)). All parameters were set to default. A phylogenetic tree was constructed in MEGA for the above sequences, using the neighbor-joining (NI) method and setting the Bootstrap to 1000. NJ trees were visualized in Chi-plot (https://www.chiplot.online/ (accessed on 16 July 2024 Beijing time)) [22]. Other parameters were set to default. The gene structure was visualized in TBtools (v2.096) using the annotation files in the genome, and the sequence motif was predicted in MEME (https://meme-suite.org/meme/ (accessed on 16 July 2024 Beijing time)), setting the number of predictions to 10. Other parameters were set to default. Protein structures were homology modeled using SWISS-MODEL (https://swissmodel.expasy.org/ (accessed on 1 July 2024 Beijing time)) and visualized in Pymol software (v3.0.0). 

### 2.3. Gene Localization and Collinearity Analysis

The location of genes on chromosomes was visualized in TBtools (v2.096) using the annotation information in the genomes (GFF3). In addition, the genomes of *Arabidopsis thaliana* and rice were downloaded from Ensembl (http://plants.ensembl.org/index.html (accessed on 21 June 2024 Beijing time)) and NCBI, and TBtools (v2.096) was used to perform genome alignment for the three species and to calculate collinearity relationships between the genomes (e < 10^−5^).

### 2.4. Promoter Analysis and Expression in RNA-Seq

Based on the genomic information, the sequence 2000 bp upstream of the gene start codon ATG was extracted, and *cis*-regulatory elements were predicted in PlantCARE (http://bioinformatics.psb.ugent.be/webtools/plantcare/html/ (accessed on 11 July 2024 Beijing time)). The results were visualized in TBtools after removing certain elements, such as TATA-box. The expression analyses were performed by downloading PRJNA255452 [23], PRJNA550274 [24], PRJNA369332 [25], and PRJNA611733 [26] from NCBI’s public database SRA (https://www.ncbi.nlm.nih.gov/sra/ (accessed on 26 June 2023 Beijing time)) by referring to previous studies [20]. Samples from these transcriptomes include bud differentiation, bud processing, fruit development, and chilling requirements. Transcriptomes were assembled using Trinity, annotated using the ‘Tieton’ v2.0 genome as a reference, and FPKM values were calculated for each gene using Hisat2 and Stringtie (v2.2.0) software. The collated data were plotted in TBtools (v2.096) as a heat map of expression, and the expression was calculated using Log_2_^(FPKM+1)^.

### 2.5. Gene Expression under Different Treatments

The experimental materials in this study were collected from Wudang District, Guiyang City, Guizhou Province, and the variety was “Sametto”. Tissue-specific expression materials were dormant flower buds, blooming flowers, young fruits, and ripening fruits, which were frozen in liquid nitrogen and stored in a −80 °C refrigerator after collection. The annual branches were collected and treated with low-temperature cooling at 4 °C, 100 mM NaCl, and 10 µM ABA, respectively. Samples were collected at 1, 3, and 6 h after treatment for preservation and reserve. For the low-temperature treatment, the temperature was set at 4 °C, light at 20,000 lx, and humidity at 80% in an artificial climate chamber. Furthermore, 25 °C was set as the control, and other conditions remained unchanged. For NaCl and ABA treatments, in an artificial climate chamber, we set the temperature at 25 °C, light at 20,000 lx, and humidity at 80%, and branches were inserted into 100 mM NaCl and 10 µM ABA for the treatments, respectively. The ddH_2_O was set as a control and other conditions remained unchanged. All the above samples and treatments were set in 3 replications. RNA extraction was performed on all samples using a kit from Omega (Norcross, Georgia) according to the instructions. The first strand synthesis of cDNA was performed using reagents from GenStar (Beijing, China). qRT–PCR experiments were performed using the synthesized cDNAs and PowerUp^TM^ SYBR^TM^ Green Master Mix (Thermo Fisher Scientific, Waltham, MA, USA) to investigate the expression patterns of *PavWHY1* and *PavWHY2*. The CDS sequences in the genome were utilized for specific primer designs with amplification products ranging from 100–300 bp, and primer specificity was tested in NCBI after completion of the primer design. Specific primers (Table S1) were designed using Primer 5 and submitted to Shanghai Biotech for synthesis (Shanghai, China, https://www.sangon.com/ (accessed on 28 June 2024 Beijing time)). qRT–PCR experiments were performed at 95 °C for 30 s of denaturation, 60 °C for 30 s of annealing, and 72 °C for 30 s of extension, using the *EF1-α2* gene as an internal reference [7], and all experiments were repeated three times. Tissue-specific expression was calculated using the expression in dormant flower buds as a control for relative expression. Relative expression was calculated in Excel (Microsoft 365, Redmond, Washington, DC, USA) using the 2^−ΔΔCt^ method; significance was calculated in SPSS26 (IBM, Armonk, NY, USA) and visualized in Origin 2021 (OriginLab, Northampton, MA, USA).

### 2.6. Promoter Cloning and Activity Analysis

Primers were designed for PCR amplification of sequences in *PavWHY1* and *PavWHY2* with reference to the sequences in the genome. For the integrity of the promoter amplification, the length of the amplification product was about 2000 bp, and the promoter region was upstream of the start codon ATG. Genomic DNA was extracted using Plant Genome Extraction Kit (Tiangen, Beijing, China) with reference to the instructions. The promoter sequences of *PavWHY1* and *PavWHY2* were ligated into the pGreenII-0800 vector (Saved by Guizhou University, Guiyang, China) using seamless cloning, respectively, and transformed into *Agrobacterium tumefaciens* GV3101 sensory state (containing pSoup plasmid, Sangon, Biotech, Shanghai, China). After expanding the culture and enriching the bacterium, the OD_600_ was adjusted to 1.0, injected into the abaxial surface of tobacco leaves, and incubated for 3d under light protection. The luminescence signals were observed in PlantView (BLT, Guangzhou, China).

### 2.7. Protein Interaction Prediction

For PavWHY1 and PavWHY2 protein interactions, predictions were made in STRING (https://cn.string-db.org/ (accessed on 11 July 2024 Beijing time)) based on protein interactions in peach (*Prunus persica*). The prediction results were visualized in Cytoscape (https://cytoscape.org/ (accessed on 11 May 2024 Beijing time)).

## 3. Results

### 3.1. Two WHY Members Exist in the Sweet Cherry Genome

After a genome-wide alignment of three *Arabidopsis thaliana* and two rice (*Oryza sativa*) WHY proteins against the sweet cherry genome v2.0 and the identification of conserved domains in CDD and Pfam, two WHY gene members were finally identified in the sweet cherry genome. They were named *PavWHY1* and *PavWHY2*, based on homology to WHY proteins in *Arabidopsis thaliana*, respectively. Subsequently, the molecular characterization of these two genes was calculated and predicted (Table 1). The amino acid weight is 29.91 kDa (PavWHY1) and 25.22 kDa (PavWHY2), and their pI values are both greater than 7, suggesting a prevalence of basic amino acids in the sequence. The instability indexes (II) of the proteins encoded by these two genes are both greater than 40, suggesting that they are unstable proteins and may be more susceptible to changes in physical and chemical properties and biological functions. The aliphatic index (AI) is 71.03 or 79.31, and the grand average of hydropathicity (GAH) is less than 1, indicating a hydrophilic protein. Prediction of subcellular localization indicates that these two proteins are expressed in plastids, while PavWHY2 may also be present in mitochondria. This information can provide a reference for further research on gene function.

### 3.2. Gene Structure of PavWHYs

**Figure 1:**
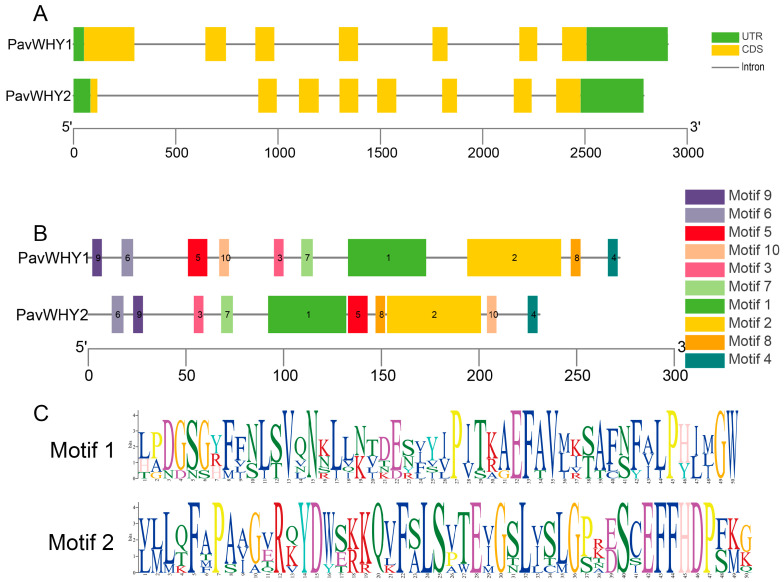
Gene structure and conserved motifs of sweet cherry Whirly gene members. (**A**) Green boxes are non-coding regions (UTR), yellow squares are exon coding sequence (CDS), and black lines are introns. The bottom scale indicates the length of the gene. (**B**) The 10 motifs of PavWHYs, distinguished by different colored blocks. The bottom scale indicates the length of the protein. (**C**) Logos of Motif 1 and Motif 2. The height of an amino acid indicates the conservation of its position.

The study of gene structure allows for the exploration of the conserved nature of these genes and their possible biological functions. Analysis of the gene structures of *PavWHY1* and *PavWHY2* revealed that they have six or seven introns (Figure 1A). The number of introns may often be related to the expression of the genes, suggesting that these genes may be expressed at higher levels in the cell [27]. Since there were only two protein sequences, the conserved motifs of the sweet cherry WHY protein were predicted at the same time as Whirly in *Arabidopsis thaliana* and rice (Figure 1B). Of the ten motifs found, Motifs 1 and 2 were the most conserved (Figure 1C). These results suggest that the WHY gene family is relatively conserved across the plant kingdom.

### 3.3. Phylogenetic and Sequence Characteristics of Whirly

**Figure 2:**
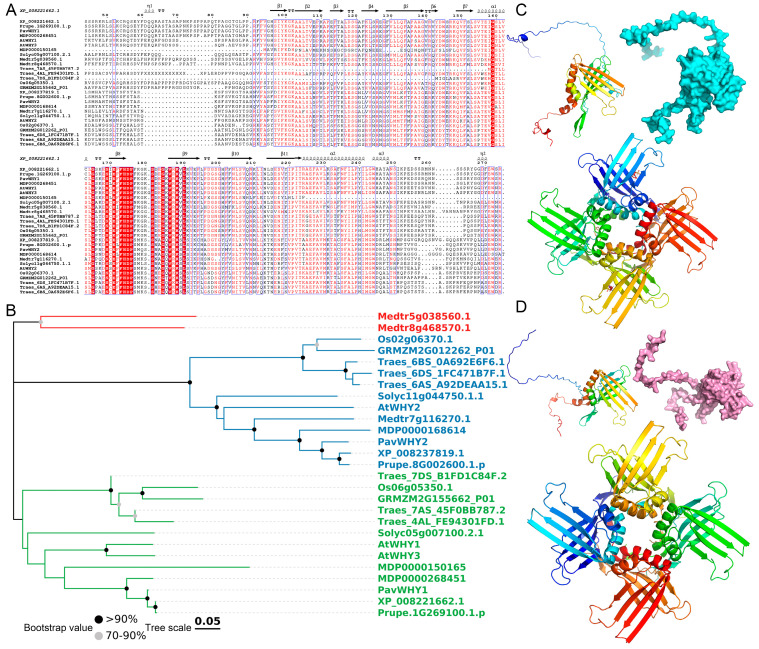
Protein sequence alignment, phylogenetic tree, and tertiary structure. (**A**) Sweet cherry, *Arabidopsis thaliana*, rice, tomato, apple, peach, wheat, thistle alfalfa, plum, and maize WHY protein sequence alignment. The red background indicates that the amino acids are completely conserved in this position. Note: *Arabidopsis thaliana* (AtWHY1, AtWHY2, AtWHY3); rice (Os02g06370.1, Os06g05350.1); tomato (Solyc11g044750.1.1, Solyc05g007100.2.1); apple (MDP0000150165, MDP0000168614, MDP0000268451); peach (Prupe.1G269100.1.p, Prupe.8G002600.1.p); wheat (Traes_6DS_1FC471B7F.1, Traes_7AS_45F0BB787.2, Traes_7DS_B1FD1C84F.2, Traes_4AL_FE94301FD.1, Traes_6AS_A92DEAA15.1, Traes_6BS_0A692E6F6.1); thistle alfalfa (Medtr5g038560.1, Medtr7g116270.1); plum (XP_008237819.1, XP_008221662.1); maize (GRMZM2G012262_P01, GRMZM2G155662_P01). (**B**) Phylogenetic tree of WHYs proteins. Different colors distinguish subcategories. (**C**,**D**) Protein tertiary structure of PavWHY1 and PavWHY2.

Homology of genes can provide a basis for probing the function of genes. To further understand the evolutionary relationships of PavWHYs, all WHY protein sequences from sweet cherry, *Arabidopsis thaliana*, rice (*Oryza sativa*), tomato (*Solanum lycopersicum*), apple (*Malus* × *domestica*), peach (*Prunus persica*), wheat (*Triticum aestivum*), thistle alfalfa (*Medicago sativa*), plum (*Prunus salicina*), and maize (*Zea mays*) were utilized for sequence comparison, and the results were used for phylogenetic tree construction (Figure 2). The results show that these sequences are very conserved in their structural domains, and that these conserved positions together constitute the typical lamellar structure of WHY proteins (Figure 2A). In detail, a blade-like structure is formed from β3 to β11, and the middle β7 and β8 are connected by an α helix. Furthermore, the evolutionary relationship of these sequences was analyzed, and the NJ tree showed that these proteins were divided into two categories (Figure 2B). *Arabidopsis thaliana AtWHY2* and other members are grouped into one category, and *AtWHY1/3* and other members are grouped into another category. Each of these two subclasses includes members of dicotyledonous and monocotyledonous plants, respectively. This phenomenon once again shows that WHY proteins are very conserved, and members of the two subclasses are equally conserved. The genes of sweet cherries, apples, and plums are on the same clade, which is consistent with the fact that they are all members of the Rosaceae family. It also indicates that the WHY genes are more conserved in these species and may share the same ancestral gene. It is worth noting that the two WHY members in *Medicago truncatula* are not in the two subclasses, but that they are clustered into a separate class. In addition, the protein tertiary structures of two WHY members in sweet cherry were predicted (Figure 2C,D). The results showed that they all have a typical blade-like extended structure and rely on the α-helix to form a homotetramer.

### 3.4. Chromosomal Location and Collinearity of PavWHYs

**Figure 3:**
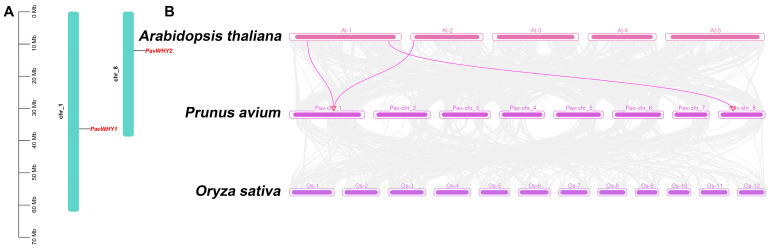
The distribution of sweet cherry WHY gene members on the chromosome (**A**) and the collinear relationship (**B**). Purple connecting lines indicate covariance between WHY genes.

Furthermore, we analyzed the chromosomal locations of the two WHY proteins in sweet cherries. The results showed that *PavWHY1* was on chromosome 1 and *PavWHY2* was on chromosome 8 (Figure 3A). The distribution of genes on chromosomes may be related to gene expression or evolution, which requires more research to prove in the future. In order to explore the homology and evolutionary relationship between sweet cherry WHY and proteins in other species, collinearity analysis of the genomes of sweet cherry, *Arabidopsis thaliana*, and rice was conducted (Figure 3B). The results showed that the *PavWHY* genes only had a collinearity relationship with members in *Arabidopsis thaliana*, but not in rice. Among them, *PavWHY1* has a collinearity relationship with *Arabidopsis thaliana AtWHY1* and *AtWHY3*, indicating that these three genes may be homologous genes. *PavWHY2* and *AtWHY2* are a collinear gene pair, suggesting that they have high evolutionary homology.

### 3.5. Promoter Cis-Acting Elements and Expression Profiles of PavWHYs

**Figure 4:**
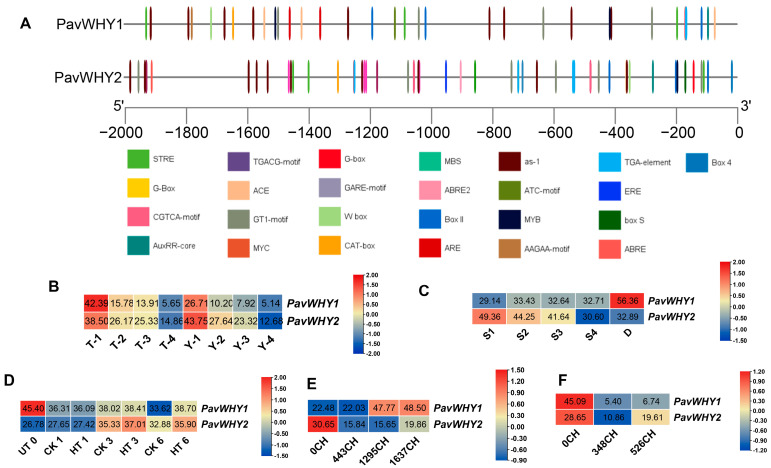
Promoter prediction and expression of PavWHYs in the transcriptome. (**A**) Predicted results of 2000 bp sequences upstream of the ATG of *PavWHY1* and *PavWHY2* genes in PlantCARE. Different colors indicate different *cis*-regulatory elements. (**B**) Expression of PavWHYs in the transcriptome for fruit development, “T” for Tieton variety and “Y” for 13–11 cultivar. (**C**) Expression of PavWHYs in the transcriptome of flower bud differentiation. (**D**) Expression of PavWHYs in the transcriptome of hydrogen cyanamide-treated flower buds. (**E**) Expression of PavWHYs during chilling requirement in the “Cordia” variety. (**F**) Expression of PavWHYs during chilling requirement in the ‘Royal Dawn’ variety.

The 2000 bp sequence upstream of PavWHYs was extracted from the sweet cherry genome to predict *cis*-acting elements. The results show that there are multiple regulatory elements, including abiotic stress, growth and development, and hormonal response (Figure 4A). In addition, there are a large number of light-responsive elements, suggesting that these two genes may be related to photosynthesis. Predictive analysis of *cis*-acting elements showed that PavWHYs may have multiple biological functions and play an important regulatory role in various life activities of sweet cherry. Subsequently, the expression of these members was analyzed in RNA-seq. Both *PavWHY1* and *PavWHY2* showed downregulated expression during fruit development in the two cultivars (Figure 4B). A trend of downregulation in expression was consistently observed throughout the four periods, implying that their expression was suppressed during fruit development. During flower bud differentiation, the expression of *PavWHY1* showed a slight upregulation change, and the expression of *PavWHY2* showed a downregulation trend, especially in the fourth stage (S4) (Figure 4C). Interestingly, *PavWHY1* shows a high level of expression in dormant flower buds, implying that it may have important functions for the hibernation of flower buds. In addition, when using hydrogen cyanamide to deal with flower buds, the expression on the first and third day after treatment was not changed significantly, and the expressions of the two genes on the sixth day of the treatment were increased to varying degrees (Figure 4D). The expression of *PavWHY1* was upregulated at a later stage during the accumulation of chilling requirements in “Cordia”, and the expression of *PavWHY2* was downregulated and then slightly upregulated throughout the process (Figure 4E). During the chilling requirement process in “Royal Dawn”, both *PavWHY1* and *PavWHY2* showed a decrease in transcription levels, but *PavWHY2* was slightly upregulated in the later stage (Figure 4F). These expression results imply that sweet cherry WHY has multiple functions and plays an important role in the regulation of growth and development and cold stress.

### 3.6. Tissue-Specific Expression of PavWHYs

**Figure 5:**
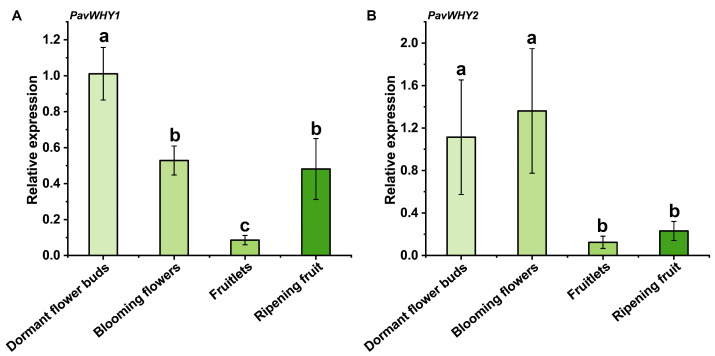
Expression of PavWHYs in dormant flower buds, blooming flowers, fruits, and ripening fruit in sweet cherry ‘Sametto’. (**A**) is the expression of *PavWHY1*, and (**B**) is the expression of *PavWHY2*. The expression level was calculated using the 2^−ΔΔCt^ method, with *EF1-α2* as the internal reference gene and the expression in dormant flower buds as the control. Data represent one-way ANOVA statistics with three replications, and different letters indicate significance at *p* < 0.05.

To further investigate the expression pattern of sweet cherry WHY genes in different tissue growth stages, qRT–PCR analysis was performed on four different tissues (Figure 5). The results showed that the expression of *PavWHY1* and *PavWHY2* was downregulated in these tissues relative to dormant flower buds. *PavWHY1* was downregulated in flowers and young fruits, and slightly upregulated in ripening fruit, but remained lower than that of dormant flower buds (Figure 5A). *PavWHY2* showed no significant change in the expression pattern of dormant flower buds and full blooming flowers but showed downregulated expression in both fruit developmental periods (Figure 5B). qRT–PCR results were in general consistent with the transcriptome results for fruit development, implying that the functions of these two genes were repressed during fruit development.

### 3.7. Expression of PavWHYs during Cold Stress Treatment

**Figure 6:**
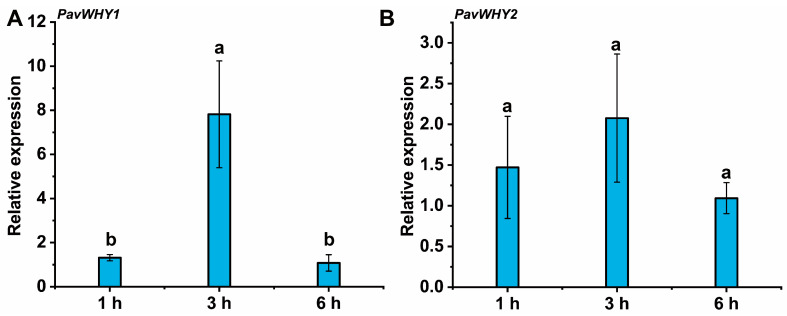
Expression of PavWHYs during cold stress treatment in leaves. (**A**) is the expression of *PavWHY1*, and (**B**) is the expression of *PavWHY2*. Data represent one-way ANOVA statistics with three replications, and different letters indicate significance at *p* < 0.05.

RNA-seq indicated that PavWHYs were differentially expressed during the two occurrences of cold storage [24], and we further explored the response patterns of these two genes under low-temperature treatment. The results showed that the expression of *PavWHY1* increased in leaves at 3 h after treatment, and the transcript level decreased at 6 h (Figure 6A). *PavWHY2* showed no significant difference among the three time points sampled (Figure 6B). The changes in expression at low temperature implied that *PavWHY1* responded to low-temperature treatment and may have a regulatory function in low-temperature stress.

### 3.8. Expression of PavWHYs during NaCl Treatment

**Figure 7:**
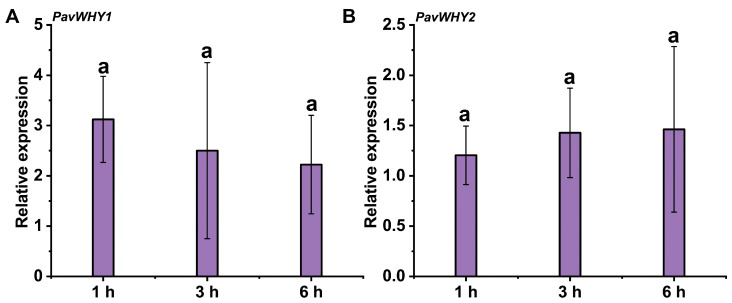
Expression of PavWHYs during NaCl treatment in leaves. (**A**) is the expression of *PavWHY1*, and (**B**) is the expression of *PavWHY2*. Data represent one-way ANOVA statistics with three replications, and different letters indicate significance at *p* < 0.05.

Furthermore, the expression of these two genes was investigated under NaCl treatment. The results showed that there was no significant difference in the expression of *PavWHY1* and *PavWHY2* after treatment with 100 mM NaCl (Figure 7). This suggests that sweet cherry WHY genes may have other functions than salt stress or that they may fail to respond to the stress in a short treatment.

### 3.9. Expression of PavWHYs during ABA Treatment

**Figure 8:**
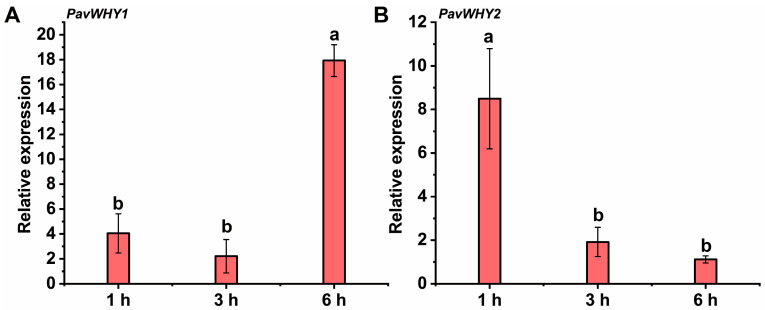
Expression of PavWHYs during ABA treatment in leaves. (**A**) is the expression of *PavWHY1*, and (**B**) is the expression of *PavWHY2*. Data represent one-way ANOVA statistics with three replications, and different letters indicate significance at *p* < 0.05.

Due to the presence of abscisic acid-responsive *cis*-elements in the promoter regions of *PavWHY1* and *PavWHY2*, we further verified the expression patterns of these two genes after ABA treatment (Figure 8). The results showed that the transcript level of *PavWHY1* increased at 6 h after treatment, with no significant change at 1 h and 3 h (Figure 8A). It is implied that *PavWHY1* was up-regulated in response to exogenous ABA treatment. Different from *PavWHY1*, the expression level of *PavWHY2* showed a down-regulation trend with treatment (Figure 8B). It indicated that the expression of *PavWHY2* was suppressed during ABA treatment, suggesting a possible regulatory role of its function in the ABA signaling pathway. The expression patterns of the two genes in ABA treatment suggest that they may have important functions in the hormone signaling regulatory network.

### 3.10. The Promoters of PavWHY1 and PavWHY2 Were Active

**Figure 9:**
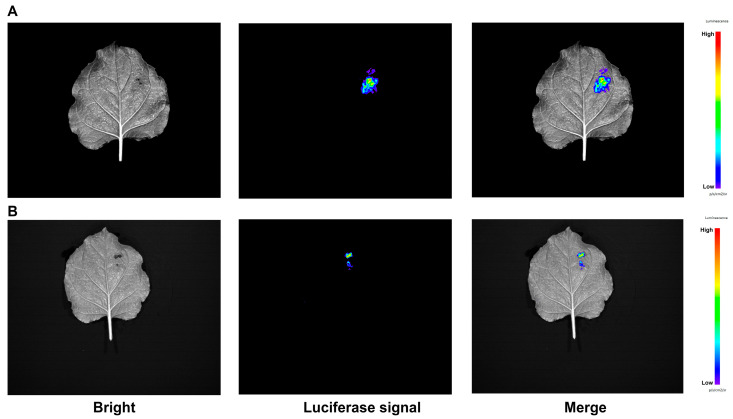
*PavWHY1* and *PavWHY2* promoters drive luciferase expression in tobacco leaves. Luminescence signals after transient transformation of tobacco by recombination of the promoters of the two genes into the pGreenII-0800 vector and incubation for 3 d in light protection. (**A**) Promoter activity of *PavWHY1*. (**B**) Promoter activity of *PavWHY2*.

Predictive analysis of the promoters of *PavWHY1* and *PavWHY2* revealed the presence of a large number of *cis*-regulatory elements and changes in expression under cold stress and ABA treatment, which implied that their promoters were more active. To verify the activity of these two promoters, their promoter sequences were ligated into the pGreenII-0800 vector, respectively, and transiently expressed in tobacco (Figure 9). *Agrobacterium tumefaciens* containing a recombinant plasmid with two gene promoters was able to show a luminescent signal catalyzed by luciferase sodium salt after transient expression in tobacco. The results showed that the promoters of both *PavWHY1* and *PavWHY2* were sufficient to drive the expression of luciferase, which emits light in response to substrate. These results indicate that the promoters of *PavWHY1* and *PavWHY2* are biologically active, suggesting that they are more active in vivo.

### 3.11. Interaction of PavWHY1 and PavWHY2 with Other Proteins

**Figure 10:**
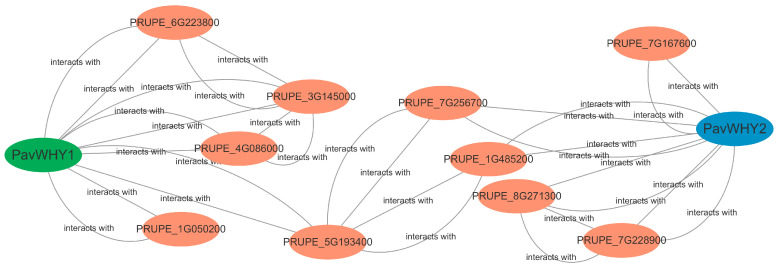
Prediction of protein interactions of PavWHY1 and PavWHY2 based on peach in STRING. PavWHY1 is indicated using green color and PavWHY2 is indicated using blue color.

As genes exercise their biological functions, they often interact with other proteins, and such interactions may be related to modification, processing, and protein activity. Furthermore, we analyzed the predicted interactions of the two WHY proteins in sweet cherry in STRING based on protein interactions in peach (Figure 10). The results show that PavWHY1 and PavWHY2 interact extensively with other proteins, but they do not interact directly. In detail, PavWHY2 interacts with plasma membrane fusion protein PRUPE_7G256700, which in turn interacts with the gibberellin-regulated family protein PRUPE_5G193400, which interacts with PavWHY1. In addition, PavWHY1 and PavWHY2 interact with other proteins, respectively, forming a complex regulatory network. This prediction implies that the function of WHY proteins in sweet cherry may be variable, and these interactions need to be proved by more in-depth studies to be resolved in the future.

## 4. Discussion

Advances in sequencing technology have resulted in high-quality genomes for many plants, which have allowed the identification of gene family members in many plants. WHY gene members have been identified and studied in many plants, such as wheat [28], tomato [29], and grape [30]. The WHY gene signature is present in angiosperms in addition to some plastid-containing algae [11]. A gene homologous to WHY1 has been found in unicellular green algae (*Chlamydomonas reinhardtii*) and hepatica (*Klebsormidium flaccidum*), but not in the cyanobacterium *Synechococcus elongates* or in *Ceratocystis paradoxa* and *Cyanidioschyzon merolae*, suggesting that this gene, which is homologous to WHY, may be a component of the green algal plastid [31]. Studies of collinearity relationships between monocot and dicot genomes indicate that WHY genes may have existed before the divergence of dicots and monocots. Combined with the phylogenetic relationship, these members are each evolutionarily conserved after the differentiation of monocots and dicots. In the plant kingdom, most species contain two WHY proteins localized in the plastid or nucleus [8,32]. *PavWHY1* and *PavWHY2* were predicted to be mainly localized in the plasmodesmata in this study, and further studies are needed to provide proof whether these two proteins are localized in the nucleus or crosstalk with the nucleus. Changes in the localization of WHY may occur during certain specific processes. During leaf senescence in *Arabidopsis thaliana*, AtWHY2 is triply localized in mitochondria, plastids, and the nucleus, and this alteration has a key role in the allocation of carbon from mother to offspring [33]. As well as being predicted to be in plastids, *PavWHY2* in this study may also be localized in mitochondria (Table 1), suggesting homology to *AtWHY2* and possible functional similarity. Although there are three WHY members in *Arabidopsis thaliana*, *AtWHY1* and *AtWHY3* share 77% sequence similarity, suggesting that these two genes may be derived from the same ancestral gene [12]. This result is also presented in the phylogenetic tree in this study, and *AtWHY1* and *AtWHY3* are on the same clade (Figure 2). It has been shown that plastid-localized proteins have functional redundancy and that mutants do not have significant phenotypic changes or are expressed only under certain specific circumstances [10]. For the two WHY genes in this study, the prediction of their tertiary structures showed that they are similar and capable of forming tetramers (Figure 2). Whirly proteins are regulators of the second pathway (the microhomology-mediated break-induced replication pathway) in chloroplasts and mitochondria [34,35]. The tetrameric Whirly proteins bind single-stranded DNA (ssDNA), and the tetramers are further assembled into tetrameric hexamers (i.e., 24-oligomers) upon the binding of long DNA molecules, a process that is dependent on K67-mediated tetramer–quadruplex interactions [34].

In the present study, PavWHYs showed different expression patterns in tissues, implying that the functions of these two genes are multifaceted. After years of research, the WHY genes in many species are understood to play very important roles in growth and development, abiotic stress, and other processes. Double mutations in *AtWHY1* and *AtWHY2/3* in *Arabidopsis thaliana* show a significant delay in flowering, with more angiosperms as well as a reduction in seed number, which is also accompanied by a decrease in seed viability [18]. *PavWHY1* and *PavWHY2* showed downregulated expression during four fruit developmental periods in both varieties (Figure 4), suggesting that the expression of the WHY gene family may be negatively associated with fruit development. Wheat dormancy decline and seed SA levels and NPR-independent SA signaling are transcriptionally regulated by repressor genes of phenylalanine ammonia lyase, and whirly and suppressor of *npr1* inducible1 genes, respectively, which are important in synergistic and antagonistic interactions among phytohormones [35]. The transcript levels of WHYs in sweet cherry were altered during fruit development and after ABA treatment, suggesting that their function may be modified, suggesting that these genes may have important functions in growth and hormonal signaling. A great deal of future genetic experimentation is needed to prove their possible functions.

The external environment is a huge influence during the growth and development of sweet cherry. In plants responding to environmental changes, some genes make changes in expression to adapt to the adverse effects of abiotic stress. In this study, both *PavWHY1* and *PavWHY2* showed significant changes in expression after low-temperature and exogenous ABA treatment. Changes in gene expression levels indicate altered functions of *PavWHY1* and *PavWHY2* following abiotic stress treatments. These results imply that WHY genes may have important functions in abiotic stress and hormonal signaling. Tomato (*Solanum lycopersicum*) *SlWHY2* was significantly induced by drought stress, and *SlWHY2* RNAi exhibited increased wilting, decreased fresh weight, reduced photosynthetic efficiency, accumulated more ROS than WT, and had lower AOX (alternative oxidase) activity [36]. The pattern of MeCIPK23-MeWHYs-mediated drought stress response in cassava indicated that the expression of *MeCIPK23* and MeWHYs was upregulated under drought stress conditions, and that MeWHYs directly bound to and activated the transcription of the PB element in the *MeNCED1* promoter, leading to increased ABA biosynthesis and enhanced drought stress response [37]. Barley lacking *WHY1* had delayed greening, lower chloroplast protein content in leaves than the wild type, and significantly lower levels of tricarboxylic acid cycle metabolites and GABA in leaves than the wild type [38]. Analysis of the promoters of these two WHY genes in sweet cherry revealed that their functions may be multiplexed (Figure 4), and transient expression in tobacco was also found to initiate luciferase expression (Figure 9), suggesting a role in vital activities. Sweet cherries are grown in rain-sheltered cultivation, which leads to better fruit quality. During the process of rain avoidance, there may be an effect on its respiration or photosynthesis, although this effect may be favorable [39]. The predicted subcellular localization of PavWHY1 and PavWHY2 and the correlation of their homologous genes in *Arabidopsis thaliana* in this study suggest that WHY is essential for the normal function of mitochondria or chloroplasts. Although the current understanding of the sweet cherry WHY genes is relatively preliminary, it still provides a basis for continued research in the future, so genetic experiments and extensive molecular biological validation are necessary, which will be addressed in future studies.

## 5. Conclusions

In this study, two Whirly gene members were identified in the sweet cherry genome using a whole-genome identification approach. The number of gene members is the same as in most species, and they were named *PavWHY1* and *PavWHY2* based on their homology with *Arabidopsis thaliana*. The subcellular localization of these two genes is predicted to be in the plastid, with theoretical isoelectric points greater than seven and both being hydrophilic proteins. This suggests that it may be involved in plastid-related functions. The phylogenetic relationships of WHYs in sweet cherry with other species, such as *Arabidopsis thaliana*, can be categorized into two groups and are not collinear with the rice genome. The promoter regions of these two genes contain *cis*-acting elements related to hormonal and abiotic stresses. Expression in RNA-seq suggests that *PavWHY1* and *PavWHY2* may have biological functions during bud differentiation, dormancy, fruit development, and chilling requirement accumulation. qRT–PCR experiments showed that these two genes responded to exogenous ABA and low-temperature treatments. Cloning of the promoters of these two genes and using tobacco transient expression assays showed that their promoters were able to initiate luciferase expression, demonstrating that the promoters had activity. Interaction prediction of the proteins showed that these two proteins have extensive interactions with other proteins. In this study, the WHY gene family of sweet cherry was identified and analyzed at the genome-wide level and the expression patterns and promoters of these gene members were analyzed in depth, and these results may provide a basis for further understanding of the WHY gene family and a reference in future breeding.

## Figures and Tables

**Table 1:** Information on the Whirly gene family members of sweet cherry. Including amino acid length (aa), theoretical isoelectric point (pI), instability index (II), aliphatic index (AI), grand average of hydropathicity (GAH), and subcellular localization prediction.

Gene Name	ID in Genome	aa	MW	pI	II	AI	GAH	Location
*PavWHY1*	FUN_039943-T1	272	29.91092	9.68	51.12	71.03	−0.371	Plastid
*PavWHY2*	FUN_027824-T1	231	25.21904	9.79	42.01	79.31	−0.07	Plastid, Mitochondrion

## Data Availability

Data are contained within the article and Supplementary Materials.

## Supplementary Materials

The following supporting information can be downloaded at: https://www.mdpi.com/article/10.3390/cimb46080474/s1, Table S1. Primer information in this study. Table S2. All sequence information in this study.
