# Supplementary material for: Genome-Wide Identification and Expression Analysis of the Sweet Cherry Whirly Gene Family
Source: Curr Issues Mol Biol. 2024 Jul 26;46(8):8015–30. doi: 10.3390/cimb46080474 (PMC11353091; doi:10.3390/cimb46080474)
Supplement: Supplementary file 1 [file cimb-46-00474-s001.zip › Table S1.pdf]

Table S1. Primer information in this study.

Primer name	Primer sequence	purpose
PavWHY1-F	TCTGCCCCTAACAAACCTTCTC	qRT-PCR
PavWHY1-R	TACCTGCTTTCTGCTCCAATCA	
PavWHY2-F	GTCACAGCTGCTGAATTTGCTGT	
PavWHY2-R	CACTTTTGATTGTTGCCGTCCTA	
Pro-PavWHY1-F	GGTCTACGATCACTCTGCATCACC	Pomoter cloning
Pro-PavWHY1-R	CCTTAACACTCCTCTCTCTCTCTCT	
Pro-PavWHY2-F	CCACAAGGACAGGAAGTACCCAAC	
Pro-PavWHY2-R	CTCTGCTGAACCTAAAGACCTCCG	
0800- PavWHY1-F	gtcgacggtatcgataagcttGGTCTACGATCACTCTGCATCACC	vector construction
0800- PavWHY1-R	agaactagtggatcccccgggCCTTAACACTCCTCTCTCTCTCTCT	
0800- PavWHY2-F	gtcgacggtatcgataagcttCCACAAGGACAGGAAGTACCCAAC	
0800- PavWHY2-R	agaactagtggatcccccgggCTCTGCTGAACCTAAAGACCTCCG	
