# Supplementary material for: Genome-Wide Identification and Expression Analysis of the Sweet Cherry Whirly Gene Family
Source: Curr Issues Mol Biol. 2024 Jul 26;46(8):8015–30. doi: 10.3390/cimb46080474 (PMC11353091; doi:10.3390/cimb46080474)
Supplement: Supplementary file 1 [file cimb-46-00474-s001.zip › Table S2.pdf]

Table S2. All sequence information in this study.

>PavWHY1 CDS

ATGCTGAGACTACACTCCCTATCTTCACCGGCGGTGGCTGCACAAAAGCCTAACCAAAAC
CCTAAACAGTGCTCGCCCTCTAGGGTTTTCAGCACCAACAGCTTTGGCTTTGCTCCGAGT
TTGAGCTCGTCGAGGAAGAGGCTCTCCTTGAAATGTCGCCAGTCTGAGTACTTCGACCAG
CAGAGGGCCAGCACGGCCTCTGCCCCTAACAAACCTTCTCCTCCTGCTCCACAAACTCCC
GCAGGAGGAACTGGGTTGCCGCCTAGGTTTTTTGTGGGTCACTCAATATACAAAGGAAAG
GCAGCTCTTACCGTAGAGCCCAAAGCTCCGGAGTTCACTGCTTTAGATTCAGGGGCATTC
AAACTCTCCAGGGAAGGCTTTGTTCTACTTCAGTTTGCTCCGGCAGCAGGTGTTCGTGTA
TATGATTGGAGCAGAAAGCAGGTATTCTCACTATCAGTGACAGAAATTGGAAGCCTGGTT
TGCCTTGGCTCGAAGGAGTCCATTGAATTCTTCCATGATCCTTTTAAGGGGAAAAGTGAC
GAGGGCAAGGTCAGGAAGGTGTTGAAGGTGGAGCCTCTTCCAGATGGATCTGGCCATTTC
TTTAACCTCAGTGTTCAGAACAAGCTTATCAACTTGGATGAGAGCGTTTATATTCCTATC
ACCAGAGCAGAGTTTGCAGTTCTCAAATCAGCTTTCAATTTTATCCTGCCGTACATTTTA
GGTTGGCATGCCTATGCAAACTCCATAAAACCAGAAGATTCGAGCCGTCAGAATAACTCT
AGTTCTAGATATGGAGGAGATTTTGAATGGAGCCGGTAG

>PavWHY2 CDS

ATGATGAAGGTTTTGCGCCTCTTGTCCTCCTCCAGGAACCAGTTCTCCACTAGGGGTGCT
TTATCAATGCATGCTCATACACATAGCACCAGCTTTTCAACTGCCAGTAAGAGTTTTTCC
GTTAAAGGAACTAGCGCTGATTATGTATATGCTCCTTTTCGTATCTACAAAGGAAGAGCT
GCACTCTCTTTGAGTCCTGTTCTCCCAACATTCACCAAATTGGAATCTGGAGGTCTTGTA
GTTGGTCGGAGTGGTGTTGTCATGTTGAAGTTCACTCCTGCCATTGGCGAGCGCAAGTAT
GACTGGGAAAAGAGACAGGTGTTTGCTTTATCAGCAACGGAGGTTGGCTCTCTAATAAGC
TTGGGTCCCAACGATTCTTGTGAATTTTTTCATGATCCCTCAATGCAATCAAGTAATGCT
GGTCAAGTGAGGAAGAGCTTATCAATTAAGTCTCATGCAGATGGCAGTGGCTACATGGTT
TCTTTGACTGTTGTAAACAACCTCCTCAAAACCAAAGAGAATTTCATAGTTCCTGTCACA
GCTGCTGAATTTGCTGTTATGAAGACTGCTTGCAGTTTTGCGCTGCCCCACATCATGGGT
TGGGATAGGTTGATGAGCAATATGCCGACAGGTGTAGGTGTAGGACGGCAACAATCAAAA
GTGGTTCCACAACTTTTGGATGAGTGGGATAGATGA

>PavWHY1 Protein

MLRLHSLSSPAVAAQKPNQNPKQCSPSRVFSTNSFGFAPSLSSSRKRLSLKCRQSEYFDQ
QRASTASAPNKPSPPAPQTPAGGTGLPPRFFVGHSIYKGKAALTVEPKAPEFTALDSGAF
KLSREGFVLLQFAPAAGVRVYDWSRKQVFSLSVTEIGSLVCLGSKESIEFFHDPFKGKSD
EGKVRKVLKVEPLPDGSGHFFNLSVQNKLINLDESVYIPITRAEFAVLKSAFNFILPYIL
GWHAYANSIKPEDSSRQNNSSSRYGGDFEWSR

>PavWHY2 Protein

MMKVLRLLSSSRNQFSTRGALSMHAHTHSTSFSTASKSFSVKGTSADYVYAPFRIYKGRA
ALSLSPVLPTFTKLESGGLVVGRSGVVMLKFTPAIGERKYDWEKRQVFALSATEVGSLIS
LGPNDSCEFFHDPSMQSSNAGQVRKSLSIKSHADGSGYMVSLTVVNNLLKTKENFIVPVT
AAEFAVMKTACSFALPHIMGWDRLMSNMPTGVGVGRQQSKVVPQLLDEWDR

>AtWHY1

MSQLLSTPLMAVNSNPRFLSSSSVLVTGGFAVKRHGFALKPTTKTVKLFSVKSRQTDYFE
KQRFGDSSSSPSPAEGLPARFYVGHSIYKGKAALTVDPRAPEFVALDSGAFKLSKDGFLL
LQFAPSAGVRQYDWSKKQVFSLSVTEIGTLVSLGPRESCEFFHDPFKGKSDEGKVRKVLK

VEPLPDGSGHFFNLSVQNKLVNVDESIYIPITRAEFAVLISAFNFVLPYLIGWHAFANSI
KPEETSRVNNASPNYGGDYEWNR
>AtWHY2
MMKQARSLLSRSLCDQSKSLFEASTLRGFASWSNSSTPGRGFPGKDAAKPSGRLFAPYSI
FKGKAALSVEPVLPSFTEIDSGNLRIDRRGSLMMTFMPAIGERKYDWEKKQKFALSPTEV
GSLISMGSKDSSEFFHDPSMKSSNAGQVRKSLSVKPHADGSGYFISLSVNNSILKTNDYF
VVPVTKAEFAVMKTAFSFALPHIMGWNRLTGHVNTEALPSRNVSHLKTEPQLELEWDK
>AtWHY3
MSQLLSSPPMAVFSKTFINHKFSDARFLSSHSILTSGGFAGKIIPLKPTARLKLTVKSRQ
SDYFEKQRFGDSSSSQNAEVSSPRFYVGHSIYKGKAALTIEPRAPEFVALESGAFKLTKE
GFLLLQFAPAAGVRQYDWSRKQVFSLSVTEIGNLVSLGPRESCEFFHDPFKGKGSDEGKV
RKVLKVEPLPDGSGRFFNLSVQNKLLNVDESVYIPITKAEFAVLISAFNFVLPHLIGWSA
FANSIKPEDSNRLNNASPKYGGDYEWSR
>Os02g06370.1
MQRLSRFVPSSSRRVTDLKDALWSGSLTFQHALSTFAADENTSGRKFASYTVFKGKAALS
MQPILPSFSKLESGGSRVNKNGSVMLTFFPAVGQRKYDYSKKQLFALSPTEVGSLISLGP
AESCEFFHDPSMKSSHEGQVKKSLSVTPLGNDSGYFLNITVLNNLQKTTERLSLPISKAE
FTVMRTALSFALPHILGWDQALTNHQPSPSPASKPRVERPHPDSEWER*
>Os06g05350.1
MPPPSPPLFLSLPSPPPPPLPHLLPSHRPAAALTLAPALSSRRVSSVCPVASQRHSDYFD
PRAPPPPPPRDGYGGPAYSPPAAQGGQQNGRVFSTYSIYKGKAAMSLDPRPPQFVPLDSG
AYKVVKEGFVLLQFAPAVATRQYDWTRKQVFSLSVWEMGSLLTLGPTDSCEFFHDPFKGR
SDEGKVRKVLKVEPTPDGNSRFFNLSVQNRLLNIDENIYIPITKGEFAVIVSTFNYIIPH
LMGWSTFTNSIKPEDSRAYTRPQSGPEYEWRR*
>Solyc11g044750.1.1
MMFFSRHIRNQLLHKKLSGEDVKGSIWQNAINTFAAFSTVRQDVVADAGKREGRVFAPYS
VFKGKAALSAEPRLPTFNRLDSGGVKLNRRGVIMLTFWPSVGERKYDWEKRQLFALSATE
VGSLISMGTRDSSEFFHDPSMLSSNAGQVRKSLSIKPNADGSGYFVSLSVVNNNLKTNDR
FTVPVTTAEFAVMRTAFSFALPHIMGWDRFTNRPSESISQSPSKVVPQLMEAEWDR*
>Solyc05g007100.2.1
MSVFSLSASPASGFSLNPTKTSSYLSFSSSINTIFAPLTSNTTKSFSGLTYKAALPRNLS
LTCRHSDYFEPQQQQQQLQGASTPKVFVGYSIYKGKAALTVEPRSPEFSPLDSGAFKLSK
EGMVMLQFAPAAGVRQYDWSRKQVFSLSVTEIGSIISLGAKDSCEFFHDPNKGRSDEGRV
RKVLKVEPLPDGSGHFFNLSVQNKLINLDENIYIPVTKAEFAVLVSAFNFVMPYLLGWHT
AVNSFKPEDASRSNNTNPRSGAELEWNR*
>Medtr5g038560.1
MKMLQLQPPQSYTTTNPFSVPTHSFIINTPKKSIFLRRVGPTFSLTCHHPELFHPKPFPP
PQRPQSSSSSFSSSVGELPARVHVSRSVYKGKAVLVVSPVLPKFTSSDSGTFKISKEGLM
LLQFVPSAGFRQYDWNRKQVFSLSVDEMGNLINLGARESCEIFHDPFMGRSDEGKVRKVL
KVEPLHDGSGHMFKLSVQNQLKNIDENIFIPVTKAEFAVFNSLFSFIMPYLLGWNAFADS
IKPEVNIANPRRREEDFEWNR*
>Medtr7g116270.1
MVPKMLKFSRMLHSSSRNHLLEVLYARDFSTATNNNYSAKGYTSDRIFAPYSVYKGKAAF
SLSPCLPTFTKLDSGALVVDRHGSIMMSFMPAIGERKYDWEKRQIFALSATEVGSLIAIG

PQDSCEFFHDPSMKSSNAGQVRKSLSIKPHSNGYFVSLSVVNSVLNTKDNFSVPVTTAEF
AVMKTACSFALPHIMGWDRLTNQQSSGTVSFQPKMNSQILDLEWEK*
>Medtr8g468570.1
MSHLQLHLHSQPPSLSSTSSSSFSSLKLFTNNNSFSLPFKFKPFTIRCRHSDVFNPSPSN
PPPPATTPPNNPLVGALPPRVYVGHSIYKGKAALTITPTPPKFVTLDSGAYKISRDGCLL
LQFAPSVGPRQYDWNRKQLFMLSVDEMGSVISLGARESCEFFHDPFKGGSDEGKVRKVLK
IEPFPDGSGFFFNLSVQDKIVNVDVSMNIPVSKAELSVLRSIFKYIMPYLLGWHAFANSI
NPEYSAALNNVANNANPRYGGDYEWNR*
>XP_008237819.1
MMKVLRLLSSSRNQFPTRGALSMHAYTHSTSFSTASKSFSVKGTSAHNVFASFDIFKGKA
ALSLNPVLPTFTKLESGNLVVSRRGVVMLKFTPAIGERRYDWEKKQVFALSATEVGSLIS
LGPKDSCEFFHDPSMKSSNAGQVRKSLSIKCHADGTGYLVSLTVVNNLLKTKENFIVPVT
AAEFAVMKTACSFALPHIMGWDRLMSNMPSGVGRQQSNVGGQQSKVVPQLLDEWDR
>XP_008221662.1
MLRLHSLSSPAVAAQNPKQCLASELSSRARVFSTNSFGFAPSLSSSRKRLSLKCRQSEYF
DQQRASTASAPNKPSPPAPQTPAGGTGLPPRFFVGHSIYKGKAALTVEPKAPEFTALDSG
AFKLSREGFVLLQFAPAAGVRVYDWSRKQVFSLSVTEIGSLVCLGSKESIEFFHDPFKGK
SDEGKVRKVLKVEPLPDGSGHFFNLSVQNKLINLDESVYIPITRAEFAVLKSAFNFILPY
ILGWHAYANSIKPEDSSRMNNSSSRYGGDFEWSR
>MDP0000150165
MAVRIFKDHPNLAVMEKNSSHGCSGRCAFQNEEEESQINQNFTLFSLSFLQVFSLSVTEI
GSLVSLGSKESLEFFHDPFKGKSDEGKVRKVLKVEPLPXGSGHFFNLSNHLFLEILVDIY
LHLIIP
>MDP0000168614
MLKVLRVLSSSTTKFRSHFCTRDASSMYAYTHITRFSTATQKFSVKGPSSHQVYASFDIF
KGKAALSLTPVLPTFTKLESGSLVVDRRGSVMLKFTPAIGERKYDWEKRQMFALSATEVG
ALISLGSNDSCELFHDPSMKSSNAGQVRKSLSIKPHADGSGYFVSLTVVNNLLKTRESFS
VPVTTAEFAVMKTACSFALPHIMGWDRLTNKMPAGGGGGGGGQESKAVPQLLEDSSATNC
CYISNNRKQEGEFNDFSLSDPSDKPPIPIPIPIPIPPDQQPTVDAIALVIHPNFANARLR
RWKISDTSSGCTRNLFKELVIKKGSSVANRRCSYSLPMNKFFFIALHWSDKRLFTEGRAV
EDCLTTKLKAIGQTKLHISMMDWGVMSYVTFNILANVSIRTQ
>MDP0000268451
MLRLHLLSSPATAQKPNQNPSQFLSSQLLSRARVFSTNTFGFAPSPILSRKRLSLKCRQS
EYFDQQRTSTAASPNKPSPAPPTPAGATGMAPRFYVGHSIYKGKAALTVEPKAPEFTPLD
SGAFKLSREGFVLLQFAPAAGVRVYDWSRKQVFSLSVTEIGSLVSLGSKESLEFFHDPFK
GKSDEGKVRKVLKVEPLPDGSGHFFNLSVQNKLINLDESIYIPITRAEFAVLKSAFNFIL
PYILGWHAYANSIKPEDSSRANNSGLKYGGDFEWSR
>Prupe.1G269100.1.p
MLRLHSLSSPAVAAQNPNQNPKQCLASELSSRARVFSTNSFGFAPSLSSSRKRLSLKCRQ
SEYFDQQRASTASAPNKPSPPAPQTPAGGTGLPPRFFVGHSIYKGKAALTVEPKAPEFTA
LDSGAFKLSREGFVLLQFAPAAGVRVYDWSRKQVFSLSVTEIGSLVCLGSKESIEFFHDP
FKGKSDEGKVRKVLKVEPLPDGSGHFFNLSVQNKLINLDESVYIPITRAEFAVLKSAFNF
ILPYILGWHAYANSIKPEDSSRMNNSSSRYGGDFEWSR*
>Prupe.8G002600.1.p

MMKVLRLLSSSRNQFPTRGALSMHAYTHSSSFSTASKSFSVKGTSAHNVFASFNIFKGKA
ALSLNPVLPTFTKLESGNLVVSRRGVVMLKFTPAIGERKYDWEKNQVFALSATEVGSLIS
LGPKDSCELFHDPSMKSSNAGQVRKSLSIKCHADGSGYLVSLAVVNNLLKTKENFIVPVT
AAEFAVMKTACSFVLPHIMGWDRLMSNMPTVVGGQQSKVVPQLLDEWDR*
>Traes_6DS_1FC471B7F.1
FFVVISFCSFLTSCNGCTNKFFFVLFRGVTDLKDVLWSGSLTFKHALSTSAANVDENASV
KKYASYTVFKGKAALSISPILPLFTKVESGGSRVDRNGSVMLTFFPAVGQRKYDYTKKQL
FALSPTEVGSLISLGPAESCEFFHDPSMKSSHEGQVKKSLSITPLGSDNGYFVNITVLNN
VQKTNERLSVPVTKAEFAVMRTALSFALPHIMGWDQALSTHPQSTSTSASKPRFEQPNPA
SEWDR
>Traes_7AS_45F0BB787.2
MGTLLTLGLTDSCEFFHDPFKGRSDEGKVRKVLKVEPTPDGNGRFFNLSVQNRLLNVDEN
IYIPITKGEYAVIVSTFNYIIPHIMGWSTFTNSIKPEESQPYNRPQSSPELEWRR
>Traes_7DS_B1FD1C84F.2
MIGLASRVFSLSVWEMGTLLTLGLTDSCEFFHDPFKGRSDEGKVRKVLKVEPTPDGNGRF
FNLSVQNRLLNVDENIYIPITKGEYAVIVSTFNYIIPHIMGWSTFTNSIKPEESQPYNRP
QSSPELEWRR
>Traes_4AL_FE94301FD.1
MPPPLSVSLPSPQPLSLLPRHARAPHSHSLALAQPLSTRAPPSSACSVVPARHSDYFXXX
XXXXXXXXXXPLERDPPVPGGQAGRVFASYSIYKGKAALAFDPRPPQFVPLESGAYKVAK
EGFVLLQFAPAVGPRQYDWTRKQVFSLSVWEMGTLLTLGLTDSCEFFHDPFKGRSDEGKV
RKVLKVEPTPDGNGRFFNLSVQNRLLNVDENIYIPITKGEYAVIVSTFNYIIPHIMGWST
FTNSIKPEESQPYNRPQSSPELEWRR
>Traes_6AS_A92DEAA15.1
MLRLSRFLPSTSRGVTDLKDVLWSGSLTFKHALSTSAANVDENASVKKYASYTVFKGKAA
LSISPILPLFTKVESGGSRVDRNGSVMLTFFPAVGQRKYDYTKKQLFALSPTEVGSLISL
GPAESCEFFHDPSMKSSHEGQVKKSLSITPLGSDNGYFVNITVLNNVQKTNERLSVPVTK
AEFAVMRTALSFALPHIMGWDQALSTHPQSTSTSASKPRFERPNPASEWDR
>Traes_6BS_0A692E6F6.1
VYNKFSFVLFRGVTDLKDVLWSSSLTFKHALSTSAANVDESTSARKFASYTVFKGKAALS
ISPILPLFTKLESGGSRVNRNGSVMLTFFPAVGQRKYDYTKKQLFALSPTEVGSLISLGP
AESCEFFHDPSMKSSHEGQVKKSLSITPLGSDNGYFVNITVLNNVQKTNERLSVPVTKAE
FAVMRTALSFALPHIMGWDQALSTHPQSTSTSASKPRFERPNPASEWDR
>GRMZM2G012262_P01
MIAHFLGFGASVGRNSLAPSPPPVRGARSVHLGRGDLRGDETMLRLSRFLPSACRRGFDL
KESLWSGSLTFQQAVSTAATNLDGNLSGKKFASYTVFKGKAALSIHPILPSFSKLESGGS
RVSKNGSVMLTFFPAVGQRKYDYTKKQLFALSPTEVGSLISLGPAESCEFFHDPSMKSSN
EGTVKKSLSITPLGSDSGYFVNITVVNSAERTNDRLSVPITKAEFAVIRTTLSFALPHIM
GWDQALTNHHPAPAPPASSRPTVERPHPDSEWER
>GRMZM2G155662_P01
MPPPAPLFLSLASTPPPALMPVHHPRAPQSLTLVPPVASSRKAAAVPACPVASPRHSDYF
DPRAPPPPRGDGGYGRPPNGAQDGRVFTSYSIYKGKAALSFDPRPPLFVPLDSGAYKVAK
EGFVLLQFAPAVATRQYDWTRKQVFSLSVWEIGTLLTLGPTDSCEFFHDPFKGRSEEGKV
RKVLKIEPTPDGNGRFFNLSVQNRLINVDESIYIPITKGEFAVIVSTFNYIIPHLMGWST

FVSSIKPEESRPYSRPQSTSEYEWRR
